# Medical students’ knowledge about curriculum development and implementation in public and private medical institutes

**DOI:** 10.12669/pjms.40.1.7705

**Published:** 2024

**Authors:** Sumera Ehsan, Ayesha Ayub, Ayesha Sadiq

**Affiliations:** 1 Sumera Ehsan, MBBS, MPhil, MHPE, PhD Head of Department, Health Professions Education & Research Department (HPERD), Faisalabad Medical University, Faisalabad, Pakistan; 2 Ayesha Ayub, MBBS, MME Senior Demonstrator, Health Professions Education & Research Department (HPERD), Faisalabad Medical University, Faisalabad, Pakistan; 3 Ayesha Sadiq, MBBS, MME Assistant Professor Medical, Department of Education, Aziz Fatimah Medical and Dental College, Faisalabad, Pakistan.

**Keywords:** Undergraduate, Curriculum, Medical students, Development, Implementation

## Abstract

**Objective:**

To assess the knowledge of undergraduate medical students about curriculum development and implementation.

**Methods:**

A quantitative descriptive study was conducted at a public sector institute and a private sector institute from September, 2022 to December, 2022. Simple random sampling was used and all the undergraduate MBBS students were included in study. Sample size was calculated using open epi and keeping confidence interval at 95% as 316 for FMU students and 218 for AFMDC students. Ethical approval was taken from FMU and permission to collect data was obtained from AFMDC. A Questionnaire was made and distributed among the students and data obtained was analyzed using SPSS 25.0.

**Results:**

Two hundred and forty-seven (49.4%) participants responded that the students don’t have any say in their curriculum, 400 (80%) were of the view that the students must have their opinion in the curriculum and 397 (79.4%) said that students must be involved in the process of planning and implementation of curriculum. When asked about the areas of deficiencies in the current curriculum, 101 (20.2%) pointed new skill, 84 (16.8%) indicated New information, new skills, teaching Methodology and effective teachers, 68 (13.6%) said teaching methodology while 51 (10.2%) indicated new skills and teaching methodology both. Five main themes were identified from the comments of students regarding curriculum, its development and implementation including; 1. Content revision, 2. Teaching methodologies, 3. Assessment, 4. Guidance and orientation and 5. Teacher student relationship.

**Conclusion:**

The undergraduate medical students are aware of the terms related to curriculum, its development and implementation but they are not being involved in any of this process. Content revision, clinical integration from initial years, quality assessment and faculty development are few areas that need improvement from students’ perspectives.

## INTRODUCTION

A curriculum consists of the “roadmap” or “guideline” of any given discipline.^1^ Both the philosophy of teaching of the instructors as well as of the educational institution serve as two of the principles upon which a curriculum is based.^2^ Curriculum is the expectations for what will be taught and what students will do in a program of study. It includes teacher-made materials, textbooks, and national and state standards and it needs to be evaluated from time to time.^1^ Curriculum development and evaluation is a complex process involving multiple stakeholders including representation from the administration, faculty, community and students as well.^3^ Students’ perceptions and attitudes are the best indicator of curriculum quality and evaluation is not complete without it.^4^ There is a gap found in literature about students’ role and their perception about curriculum, its development and implementation especially in our country Pakistan. This study was designed to assess the knowledge of undergraduate medical students about curriculum development and implementation.

Our objective was to assess the knowledge of undergraduate medical students about curriculum development and implementation.

## METHODS

A quantitative descriptive study was conducted at Punjab Medical College (PMC), a public sector institute affiliated with Faisalabad Medical University (FMU) and Aziz Fatimah Medical and Dental College (AFMDC), a private sector institute affiliated with University of Health Sciences, Lahore from September, 2022 to December, 2022. Simple random sampling was used and all the undergraduate MBBS from 1^st^ year to final year students were included in study. Sample size was calculated using open epi and keeping confidence interval at 95% as 316 for FMU students and 218 for AFMDC students. A twenty-five item Questionnaire was made which consisted of both open ended and closed ended questions covering the knowledge of students about curriculum and its implementation as well. Fifteen questions were designed to check their knowledge about curriculum while nine questions were to check their understanding about curriculum development and implementation process. One question was general. Total 350 questionnaires were distributed among the students of FMU and 250 were distributed among the students of AFMDC.

### Ethical Approval

was taken from ERC board of FMU number 48ERC/FMU/2022-23/7 and permission was taken from AFMDC.

### Statistical Analysis

Data obtained was analyzed using SPSS 25.0 for open ended question, manual thematic analysis was performed.

## RESULTS

Total 500 responses were received out of which 281(56.2%) were from FMU and 219 (43.8%) were from AFMDC. One hundred and twenty-two (24%) participants were male while 378(76%) were females. Three hundred and fifty-two students (70.4%) were aware that from 2023-24, medical graduates from those institutes whose curriculum is not revised and updated according to WEFM, would not be able to work in United states. Four hundred and seventy-eight students (95.6%) were familiar with the term curriculum and 423 (84.6%) stated that they knew what a curriculum is, 37 (7.4%) didn’t knew and 40(8%) were not sure what actually a curriculum is. Two hundred and forty-seven (49.4%) responded that the students don’t have any say in their curriculum while 400 (80%) were of the view that the students must have their opinion in the curriculum and 397 (79.4%) said that students must be involved in the process of planning and implementation of curriculum. Majority of the students i.e.299 (59.8%) didn’t know about the different types of curriculum and only 150(30%) stated that they are familiar about different types of curriculum.

According to 382(76.4%) participants, they have a tradition (pre-clinical and clinical) curriculum, 94(18.8%) said they have integrated modular curriculum while 24(4.8%) were of the view that they have problem based curriculum. Three hundred and twenty-three (64.6%) had no idea when their current curriculum was updated and only 79(15%) responded that they know about this. Regarding curriculum committee 215(43%) were not sure if such a committee exist in their institute while 187(37.4%) students stated that their medical school has a curriculum committee. Two hundred and thirty-nine (47.8%) students were not sure that the curriculum committee is functional while 138(27%) thought that the committee is non-functional.

Two hundred and twenty (44%) participants were not sure about existence of a supervising body to ensure quality implementation of curriculum. Two hundred and forty-three (48.6%) students stated they didn’t get proper orientation about the academic year programme at the start of academic year while 213(42.6%) stated that they get this information. Three hundred and thirty-three (66.6%) participants knew about study guides, 446(89.2%) responded that study guides are the basic right of students but 241 (48.2%) said that they were not provided with the study guides at the start of academic year and 53(10%) were not sure whether they were given the study guides or not. According to two hundred and thirty-nine students (47.8%), academic calendar was provided at the start of program while 39 students were not sure about it.

Learning outcomes were explained at the start of educational programme according to 243 (48.6%) students. When asked about that who develop curriculum, 144 (28.8%) students stated that the university makes curriculum while 116 (23.2%) said that the central body and the university develop the curriculum together (Table-I). One hundred and eighty-two (36.4%) stated that feedback was taken from them for assessment only, 108 (21.6%) voted for classroom settings, 77 (15.4%) indicated whole programme feedback, while 21 (4.2%) said that feedback was taken for assessment process and class room settings (Table-II). When asked about the areas of deficiencies in the current curriculum, 101 (20.2%) pointed new skill, 84 (16.8%) indicated New information, new skills, teaching Methodology and effective teachers, 68 (13.6%) said teaching methodology while 51 (10.2%) indicated new skills and teaching methodology both (Table-III). Five main themes were identified from the comments of students regarding curriculum, its development and implementation including 1. Content, 2. Teaching methodology, 3. Assessment, 4. Guidance and orientation and 5. Teacher student relationship.

**Table-I:** Authorities involved in curriculum development.

S. No.		Frequency	Percent
1-	Central Government body	102	20.4
2-	Central Government body, Student Council	6	1.2
3-	Central Government body, The University	116	23.2
4-	Central Government body, The University, Student Council	5	1.0
5-	Central Government body, The University, Your College	19	3.8
6-	Central Government body, The University, Your College, Student Council	19	3.8
7-	Central Government body, Your College	4	.8
8-	Student Council	12	2.4
9-	The University	144	28.8
10-	The University, Student Council	14	2.8
11-	The University, Your College	25	5.0
12-	The University, Your College, Student Council	6	1.2
13-	Your college	23	4.6
14-	Your College, Student Council	5	1.0

	Total	500	100.0

**Table-II:** Feedback of students received in different areas.

S. No.		Frequency	Percent
1-	Assessments	182	36.4
2-	Assessments, Class room settings	21	4.2
3-	Assessments, Clinical wards	14	2.8
4-	Assessments, Clinical wards, Class room settings	8	1.6
5-	Class room settings	108	21.6
6-	Clinical wards	27	5.4
7-	Whole year program	77	15.4
8-	Whole year program, Assessments	19	3.8
9-	Whole year program, Assessments, Class room settings	9	1.8
10-	Whole year program, Assessments, Clinical wards	6	1.2
11-	Whole year program, Assessments, Clinical wards, Class room settings	18	3.6
12-	Whole year program, Class room settings	8	1.6
13-	Whole year program, Clinical wards	3	.6

	Total	500	100.0

**Table-III:** Areas in curriculum that need to be improved.

S. No.		Frequency	Percent
1-	Effective teachers	12	2.4
2-	New information	49	9.8
3-	New information, Effective teachers	1	.2
4-	New information, New skills	31	6.2
5-	New information, New skills, Effective teachers	5	1.0
6-	New information, New skills, Teaching Methodology	42	8.4
7-	New information, New skills, Teaching Methodology, Effective teachers	84	16.8
8-	New information, Teaching Methodology	12	2.4
9-	New information, Teaching Methodology, Effective teachers	2	.4
10-	New skills	101	20.2
11-	New skills, Effective teachers	5	1.0
12-	New skills, Teaching Methodology	51	10.2
13-	New skills, Teaching Methodology, Effective teachers	18	3.6
14-	Teaching Methodology	68	13.6
15-	Teaching Methodology, Effective teachers	19	3.8

	Total	500	100.0

### Content

The students pointed out that they want the content to be more practical based and less theoretical. A vast majority of the students commented for early clinical exposure and also relation of basic sciences knowledge to clinical significance. Revision of curriculum according to international standards and integration to be done as per students’ view.

### Teaching Methodology

Teaching skills of faculty, use of new teaching modalities and improvement in methods of teaching were the areas that need to be worked upon according to students. The 1^st^ year and 2^nd^ year students specially commented about tutorials and majority of them stated that they must be interactive and student centered.

### Assessment

The students also advocated continuous assessment throughout the academic year. The quality of MCQ should be according to standards. Questions of higher cognition level and scenario based SEQs must be include.

### Guidance and Orientation

The participants also want that guidance about the course work, assessment and teaching and learning strategies must be given to them at the start of academic year. Information about vacations and extra-curricular activities must also be made available to them as well.

### Teacher Student Relationship

Participants of the study highlighted the gap between the students and teachers. They commented that there must be a strong, balanced and friendly relationship between the teachers and students. A lot of students feel hesitant talking to the teacher and communicating about their problems.

## DISCUSSION

Curriculum change is one of the most important and significant project undergoing in medical institutes of our country. Sensitization about the change is the first step in this process and without it, implementation of the new system cannot be possible. Every stakeholder must be taken on board and involved in the process to increase acceptance and quality output.^5^ This study was conducted to get an insight of what one of the stake holders i.e. students know and think about their present curriculum and the ongoing process of revision.

Majority of the students included in this study were of view that students must be involved in the process of curriculum development but they are not being made a part of this process. Involvement of students in curriculum development is not a very probed matter overall but it has been proven that student involvement in program development leads to high level of engagement by the students. A study in which students were included as module co-directors in curriculum development, 90% of faculty and 94% of students responded that the curriculum development process was benefited from students’ participation and well accepted practically applicable modules were developed.^6^

A student Curricular Board at the University of Illinois College of Medicine-Chicago provides a structured platform for student involvement in curriculum development and has shown that these students show increased interest in academic medicine as a carrer.^7^ To overcome the challenges involved in any process and project involving change, a partnership between all stake holders including students is mandatory.^8^ Now a days, medical students are using different resources including text books, notes and e learning tools.^9^ The main thing that matters is to identify the required content and suitable available resources at particular academic point. For this purpose, study guides are very helpful and include all the required information for students.^10^ moreover, study guides help in shifting from teacher centered to student centered learning.^11^ The participants of our study were aware of the importance of study guides but majority of them said that they were not provided with any guide and orientation about the programme as well.

Students of our study advocated the need to revise the curriculum and update the content based on clinical relevance and skill acquisition. It is proven that the content taught, how and when it is taught during the educational journey can significantly impact the clinical reasoning of graduating medical students.^12^ Vertical integrations between the clinical and basic science in medical curricula promote professional identity formation and engagement in students.^13^ same was proposed by the students of our study.

Improvement in teaching methodologies and faculty development was another valued point in our study. Without Faculty sensitization and development, curricular reforms cannot be implemented successfully and can lead to disaster.^14^ Faculty development programmes before and during curricular reforms must be carried out so that the new changes can be implemented truly and fully.^15^ A gap between students and faculty was highlighted by the participants of our study as well. To achieve academic outcomes, a strong student-faculty relationship build of trust and respect is needed.^16^ Students’ motivation and engagement is highly dependent of student-teacher relationship.^17^

### Limitation of the study

Faculty perspective is not taken in this study and only students were explored. More in-depth research should be carried out based on these initial results of what the student’s actual want to change in content, teacher-student relationship and assessment. Further, how much integration is required and what is their perspective about it must be probed.

## CONCLUSION

The undergraduate medical students are aware of the terms related to curriculum, its development and implementation but they are not being involved in any of this process. Students are well aware of the need to upgrade the current curriculum but are not sure whether this is happening in reality or not because of lack of communication. Content revision, clinical integration from initial years, quality assessment and faculty development are few areas that need improvement from students’ perspectives.

### Authors Contribution:

**SE:** Conceived, designed and did final editing of manuscript.

**AA:** Did data collection, analysis, and manuscript writing, is also responsible for the integrity and accuracy of the study

**AS:** Did data collection and review.
